# Long-Term Clinical Outcomes of Kidney Transplantation from Hepatitis B Surface Antigen (HBsAg)-Positive Donors to HBsAg-Negative Recipients: A Systematic Review and Meta-Analysis of Real-World Studies

**DOI:** 10.3390/jcm15156048

**Published:** 2026-08-04

**Authors:** Chengze Liang, Yun Luo, Saifu Yin, Turun Song, Tao Lin

**Affiliations:** 1 Department of Urology, Institute of Urology, West China Hospital, Sichuan University, Chengdu 610093, China; 2024224020186@stu.scu.edu.cn (C.L.);; 2 West China Medical School of Sichuan University, Chengdu 610093, China; 3 Kidney Transplantation Center, West China Hospital, Sichuan University, Chengdu 610093, China; 4 Organ Transplantation Center, West China Hospital, Sichuan University, Chengdu 610093, China

**Keywords:** kidney transplantation, HBV, donor-derived infection, organ shortage, meta-analysis

## Abstract

**Background**: The use of hepatitis B surface antigen-positive (HBsAg+) kidneys may expand the donor pool for transplantation. This study evaluated the incidence, risk factors, complications, organ function, and survival outcomes of transplants from HBsAg+ donors to HBsAg− recipients. **Methods**: A systematic search of five databases was conducted through 20 August 2023. Incidence was expressed with 95% confidence intervals (CIs); odds ratios (ORs) and hazard ratios (HRs) assessed risk factors and survival. **Results**: Sixteen studies were included. Donor-derived HBV infection occurred in 3.60% (21/583). Risk was higher in HBsAb-negative (OR: 6.67, 95% CI: 1.08–41.10) and HBcAb-negative recipients (OR: 12.96, 95% CI: 2.06–81.69). Abnormal liver function occurred in 35.18%, hepatitis in 3.07%, and hepatic failure in 0.84%. Liver and kidney function at 1–3 years were comparable between HBsAg+ and HBsAg− kidney recipients. Five- and ten-year graft survival (85.2%, 82.7%) and patient survival (93.0%, 91.4%) were similar to controls (*p* > 0.05). **Conclusions**: HBsAg+ kidneys can achieve acceptable long-term outcomes with appropriate antiviral prophylaxis and cautious recipient selection, though infection risk requires careful assessment. These findings supports the cautious and selective use of HBsAg-positive kidneys in appropriately selected recipients use and standardized HBV prophylaxis.

## 1. Introduction

Kidney transplantation (KT) remains the optimal and cost-effective treatment for end-stage kidney disease [1]. According to the latest American Organ Procurement and Transplant Network annual report of kidney transplantation [2], the total number of adult candidates listed for kidney transplant rose to 139,025, while the annual number of kidney transplants were 25,487. The growing disparity between the number of patients listed for transplantation and the limited number of available organs has led to significant morbidity and mortality. Addressing this pressing issue demands innovative approaches to augment the donor supply. One promising endeavor involves the exploration of donors with hepatitis B virus (HBV) infection [3]. In most transplant centers [4], HBsAg+ grafts were discarded due to concerns of donor-derived HBV infection and HBV-related complications. Globally, an estimated 316 million individuals (4.1%) are hepatitis B surface antigen carriers (HBsAg+), with varying prevalence rates across regions, such as 7.1% in the Western Pacific region and 1.1% in the European region [5]. This means that a significant portion of potential donor candidates are HBV-infected. Excluding HBsAg+ donors would aggravate organ shortage to some extent.

According to the latest Kidney Disease: Improving Global Outcomes (KDIGO) Clinical Practice Guideline and British Transplantation Society Guideline [4,6], HBsAg+ donors may be considered for HBsAg+ recipients or HBsAg− recipients with HBV immunity. Allocating HBsAg+ kidneys to HBsAg+ recipients is deemed reasonable as it obviates the need for a change in continuous antiviral therapies to prevent HBV progression. However, this allocation model did not achieve the full utilization of resources. As reported, the prevalence of HBsAg+ was estimated at 0.3–0.7% in the United States, while HBsAg+ donors accounted for only 0–0.2% among all kidney donors [2,7,8].

Then, explorations have been performed to allocate HBsAg+ kidneys to HBsAg− recipients. However, this pattern has not been put into clinical practice in most transplant centers due to arising concerns [9,10,11]. First, donor-derived HBV transmission can occur due to the direct input of HBV virions carried by the HBsAg+ kidneys and body fluid. A growing body of studies has evaluated transmission risk, ranging from 0% to 16.3% [12,13,14,15,16]. Second, HBV infection has been reported as an independent risk factor of impairing graft and patient survival [17]. Under immunosuppression, HBV infection can cause severe hepatic complications, including fulminant hepatitis and hepatic failure [6,18]. It remains unclear whether utilizing HBsAg+ kidneys would impair kidney graft function and survival rates. Thirdly, standardized antiviral prophylaxes have not been established [19]. Last, inaccurate data lead to insufficient informed consent for both physicians and patients.

Systematic reviews with meta-analysis have been considered as a cornerstone of evidence-based decision-making and clinical practice guidelines [20]. It gathers and critically appraises all relevant studies on a specific research question to summarize and synthesize existing evidence, increase the sample size and statistical power, and enhance generalizability. In the current work, we conducted a systematic literature review and meta-analysis, aiming to evaluate the incidence, risk factors, and clinical outcomes of donor-derived HBV infection from HBsAg+ donors to HBsAg− recipients.

## 2. Methods

### 2.1. Search Strategy

This systematic review with meta-analysis has been registered in PROSPERO (CRD42022385379) and performed following the Preferred Reporting Items for Systematic Reviews and Meta-Analyses (PRISMA) [21]. We performed comprehensive searches in five electronic databases (PubMed, Embase, Cochrane Library, Scopus, and Web of Science) from their inception to 20 August 2023 to identify all relevant studies involving KT from HBsAg+ donors to HBsAg− recipients. The searching utilized specific keywords as reported by previous systematic reviews [22,23,24,25], including “kidney transplantation”, “renal transplantation”, “kidney graft”, “renal graft”, “kidney transplant”, “renal transplant”, “solid organ transplantation”, “kidney donor”, “hepatitis B”, “hepatitis B virus”, “HBV”, “hepatitis B surface antigen”, “HBsAg”, “HBsAb”, “anti-HBs”, “HBeAg”, “HBcAb”, “anti-HBc”, “HBeAb”, and “anti-HBe” (Table S1). The language was limited to English. To ensure completeness, we manually searched the reference lists of eligible publications to identify any relevant articles that may not have been captured in the primary search.

### 2.2. Study Selection

Two independent reviewers conducted the initial screening of titles and abstracts. Subsequently, the same reviewers independently assessed the full-text articles. Any discrepancies were resolved through consensus or by discussion with a third reviewer to ensure robust selection criteria. The inclusion criteria were established based on PICOS: (1) Population, kidney transplantation recipients; (2) Intervention, utilizing HBsAg+ kidneys; (3) Comparison, utilizing HBsAg− kidneys; (4) Outcome, HBV infection and posttransplant outcomes. The exclusion criteria were as follows: (1) kidney transplantation from HBsAg+ donors to HBsAg+ recipients; (2) reviews; and (3) studies with sample sizes less than 5. In cases where multiple studies had overlapping populations, we prioritized the inclusion of studies with more recent data, longer follow-ups, larger sample sizes, and/or more comprehensive subgroup analysis data.

### 2.3. Data Collection and Quality Assessment

Two independent authors utilized a predesigned standardized extraction form to collect the following data: first author’s name, publication year, country, study design, sample size, demographic information, transplant characteristics, follow-up duration, posttransplant antiviral prophylactic regimens, donor and recipient HBV serology, HBV infection incidence, liver function, hepatitis, hepatic failure, as well as graft and patient survival. If relevant data were not readily accessible in the publications, the authors were contacted to obtain additional information.

To evaluate the risk of bias in the included studies, we employed the Newcastle–Ottawa scale (NOS) with three scoring domains: selection of participants, comparability of study groups, and ascertainment of outcomes of interest [26]. Studies scoring > 8 were classified as low risk, while those scoring 5–7 were classified as moderate risk, and studies with a score of 4 were classified as high risk.

### 2.4. Study Outcomes

We evaluated the incidence of HBV infection, defined as serological conversion to HBsAg, HBeAg, or HBV DNA positivity, with the understanding that isolated HBcAb conversion without HBsAg/HBeAg/HBV DNA indicates resolved exposure rather than active donor-derived infection. Second, we evaluated the risk factors of HBV infection. Third, we assessed HBcAb and HBsAb, defined as serological conversion to HBcAb and HBsAb positivity. Fourth, we evaluated HBV complications, including the incidence of abnormal liver function, hepatitis, and hepatic failure. Fifth, we evaluated liver function (alanine aminotransferase [ALT], aspartate aminotransferase [AST], and total bilirubin [TBIL]) and kidney function (creatinine and estimated glomerular filtration rate [eGFR]). Last, we evaluated long-term graft and patient survival. Of note, in this meta-analysis, donor-derived HBV infection was defined exclusively as seroconversion to HBsAg, HBeAg, or HBV DNA positivity. Isolated conversion to HBcAb alone, without concurrent HBsAg, HBeAg, or HBV DNA positivity, was not considered a donor-derived active infection and was analyzed separately as a secondary endpoint. Similarly, HBsAb seroconversion likely reflected passive antibody transfer (HBIG) or active vaccination, not active infection, and was treated as a distinct outcome.

### 2.5. Statistical Analyses

Meta-analysis was performed using R statistical software (version 4.2.2) with the package “meta”. The HBV infection incidence was pooled using a single-proportion meta-analysis with the inverse variance method. To stabilize the variance of risk estimates due to the low number of HBV infection events, we applied the Freeman–Tukey double arcsine transformation [27]. Although the Freeman–Tukey transformation stabilizes variance in proportions, the analysis of rare events (21/583) remains sensitive to the inclusion of zero-event studies and the choice of weighting method. Alternative approaches, such as the beta-binomial model or exact likelihood methods, may provide alternative estimates but were not implemented.

Sensitivity analyses were conducted by sequentially removing individual studies to evaluate the robustness of the pooled estimates. Meta-regression analyses were conducted to identify potential factors associated with heterogeneity. Contour-enhanced funnel plots and Egger tests were conducted to evaluate publication bias. Similarly, we pooled the incidence of HBV complications, HBcAb+, and HBsAb+.

The percentage with 95% confidence interval (CI) was used as an effect estimate for the incidence, odds ratio (OR) with 95%CI was used as an effect estimate for risk factors, hazard ratio (HR) along with 95%CI was used for comparing graft survival and patient survival, and mean difference (MD) was used for liver function and kidney function evaluation. Heterogeneity was evaluated by I^2^. I^2^ > 50% was considered to indicate significant heterogeneity, and a random-effect model was used; otherwise, a fixed-effect model (common effect model) was used.

Given the limited number of studies, meta-regression analyses have low statistical power and a risk of overfitting. Moreover, multiple covariates were examined without adjustment for multiplicity; thus, any statistically significant findings should be regarded as exploratory rather than confirmatory.

## 3. Results

### 3.1. Study Selection

**Figure 1:**
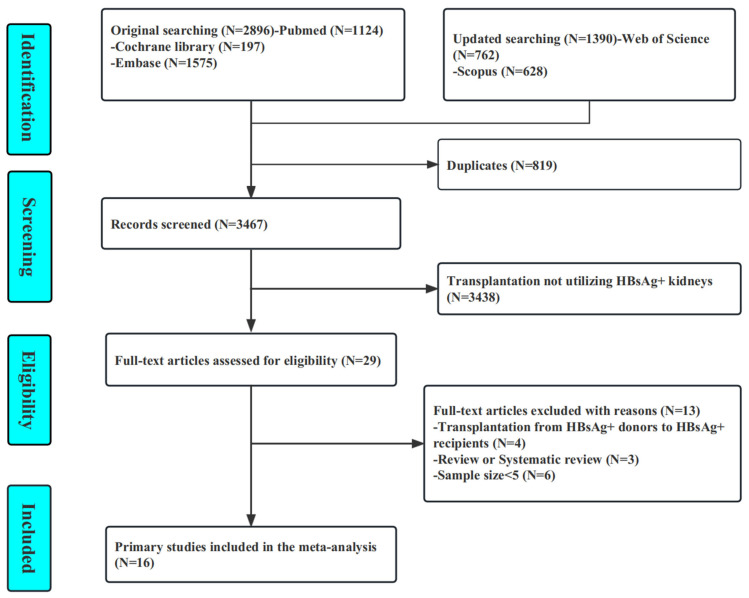
Study selection.

A total of 3467 publications were identified after removing duplicates. After screening the abstract and full text, 16 studies were finally included in this systematic review and meta-analysis [12,13,14,15,16,28,29,30,31,32,33,34,35,36,37,38] (Figure 1). The literature and reasons for exclusion are shown in Table S2.

### 3.2. Study Characteristics

These studies were published between 1993 and 2023, with 14 adopting a retrospective design (Table 1). Geographically, two studies were conducted in the USA [12,13], five in Turkey [15,16,30,34,35], five in China [14,31,32,37,38], two in Thailand [33,36], and two in France [28,29]. The sample size ranged from 7 to 144 participants. Among 16 studies, seven were designed as single-arm studies, and nine included a control group. Recipient HBsAb status was not reported in five studies, and HBcAb status was not reported in eleven studies. The median/mean follow-up duration ranged from 183 days to 137 months. Two studies used overlapping population [14,37]. The antiviral prophylactic protocols exhibited substantial heterogeneity, with varying combinations of hepatitis B immunoglobulin (HBIG) and antiviral therapy, as well as differences in dosage, frequency, and treatment duration. Regarding the bias risk of the primary studies, two studies scored 9, four scored 8, three scored 7, one scored 6, and six scored 5 (Table S3).

### 3.3. HBV Infection in HBsAg− Recipients Receiving HBsAg+ Kidneys

**Figure 2:**
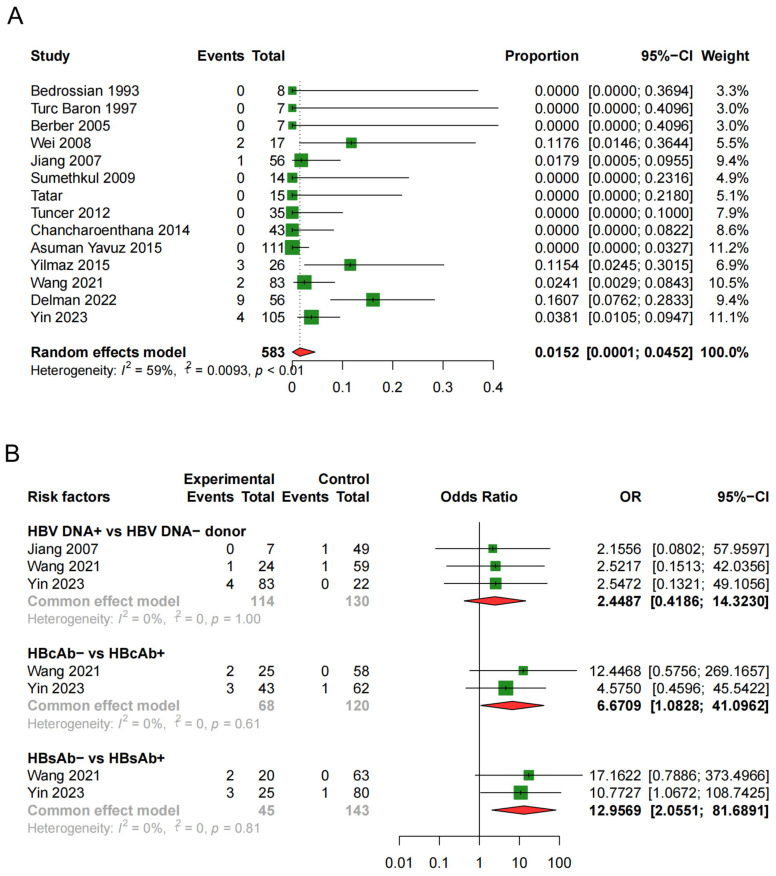
(**A**) Pooled incidence of HBV infection after kidney transplantation from HBsAg+ donors to HBsAg− recipients [12,15,16,28,29,30,31,32,33,34,35,36,37,38]. (**B**) risk factors of HBV infection [32,37,38].

Overall, 819 HBsAg− recipients from 16 studies received HBsAg+ kidneys. The incidence of HBV infection was available in 15/16 studies. The incidence of HBV infection was at 21/583 (3.60%) [12,13,15,16,28,29,30,31,32,33,34,35,36,38]. After weighting, the pooled incidence was estimated at 1.52% (95%CI: 0.01–4.52%) with high heterogeneity (I^2^ = 59%) (Figure 2A). Sensitivity analysis indicated that heterogeneity was significantly reduced after removing the study by Delman et al. (I^2^ = 28%) (Figure S1) [12]. The contour-enhanced funnel plot is shown in Figure S2. The Egger test showed no publication bias (*p* = 0.545).

Meta regression was further conducted to identify potential risk factors associated with heterogeneity, including study region (*p* = 0.032), HBV DNA status (*p* = 0.019), and HBsAb status (*p* < 0.001) (Table 2). Heterogeneity has been reduced significantly in the subgroup analyses based on meta regression (Figure S3). The incidence of HBV infection was lower if the donors were HBV DNA− (2/248 [0.81%]) without heterogeneity (I^2^ = 0%) and higher if the donors were HBV DNA+ (17/239 [7.11%]) with high heterogeneity (I^2^ = 63%). The incidence was lower in HBcAb+ recipients (1/120 [0.83%]) without heterogeneity (I^2^ = 0%) and higher in HBcAb− recipients (5/68 [7.35%]) without heterogeneity (I^2^ = 0%). The incidence was lower in HBsAb+ recipients (5/442 [1.13%]) without heterogeneity (I^2^ = 16%) and higher in HBsAb− recipients (5/45 [11.11%]) without heterogeneity (I^2^ = 0%). The incidence was 9/71 (12.68%) by enrolling studies from non-Asian regions with moderate heterogeneity (I^2^ = 33%) and 12/512 (2.34%) by enrolling studies from Asian regions with moderate heterogeneity (I^2^ = 40%).

### 3.4. Risk Factors of HBV Infection

Utilizing HBV DNA+ donors was associated with numerically higher risk of HBV infection *(n* = 3 studies, OR: 2.45, 95%CI: 0.42–14.32, *p* = 0.320) without heterogeneity (I^2^ = 0%) (Figure 2B) [14,32,38]. HBsAb− recipients had higher risk of HBV infection (*n* = 2 studies, OR: 6.67, 95%CI: 1.08–41.10, *p* = 0.006) without heterogeneity (I^2^ = 0%) [32,38]. HBcAb− recipients had higher risk of HBV infection (*n* = 2 studies, OR: 12.96, 95%CI: 2.06–81.69, *p* = 0.041) without heterogeneity (I^2^ = 0%) [32,38].

### 3.5. Outcomes After HBV Infection

Among 21 HBV infection events, 16 (76.10%) occurred within the first year. Entecavir has gradually replaced Lamivudine in recent publications. Also, with the extension of Entecavir treatment and the increase in HBIG dosage, HBV infection occurred later (Figure S4). After treatment, 14/21 (66.67%) had HBsAg clearance, while 2 recipient (9.52%) experienced hepatic failure.

### 3.6. HBcAb and HBsAb Status

Three studies reported the incidence of HBcAb (34/240 [14.17%]) [12,14,38]. After weighting, the pooled incidence was estimated at 11.20% (95%CI: 2.48–24.52%) with high heterogeneity (I^2^ = 87%) (Figure S5). Two studies reported the incidence of HBsAb (29/188 [15.43%]) [14,38]. After weighting, the pooled incidence was estimated at 15.42% (95%CI: 10.54–21.01%) without heterogeneity (I^2^ = 0%) (Figure S5).

### 3.7. Liver and Kidney Function

**Figure 3:**
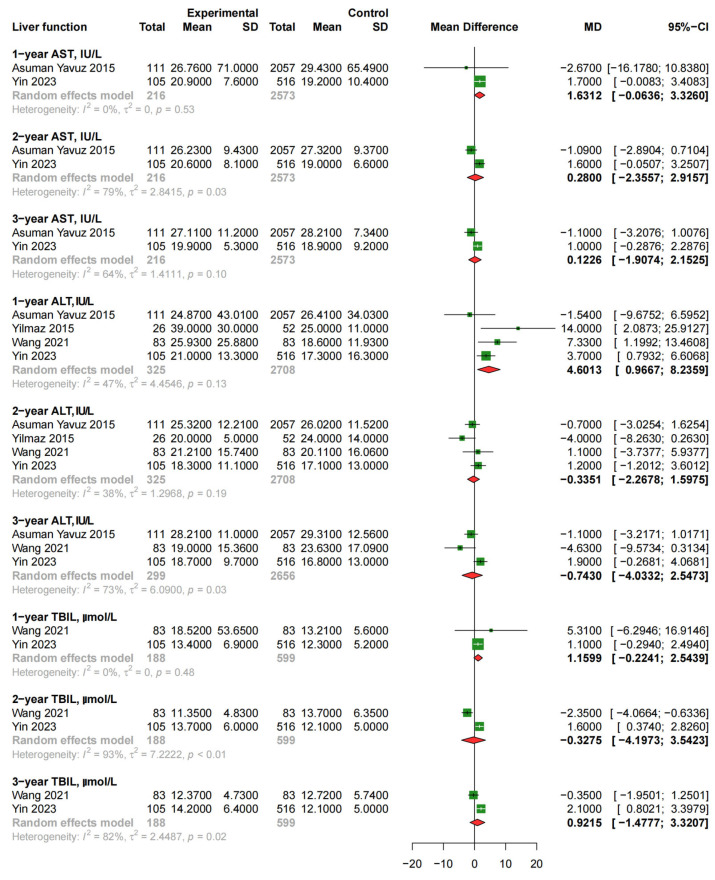
Comparison of liver function of HBsAg− recipients between utilizing HBsAg+ kidneys and utilizing HBsAg− kidneys [15,16,37,38]. ALT: alanine aminotransferase; AST: aspartate aminotransferase; TBIL: total bilirubin.

Compared with utilizing HBsAg− kidneys, utilizing HBsAg+ kidneys had similar 1-year (*p* = 0.059, I^2^ = 0%), 2-year (*p* = 0.835, I^2^ = 79%), 3-year AST levels (*p* = 0.906, I^2^ = 64%) (Figure 3). Utilizing HBsAg+ kidneys had higher 1-year ALT (*p* = 0.013, I^2^ = 47%) but similar 2-year (*p* = 0.734, I^2^ = 38%) and 3-year ALT levels (*p* = 0.658, I^2^ = 73%). Utilizing HBsAg+ kidneys had similar 1-year (*p* = 0.100, I^2^ = 0%), 2-year TBIL (*p* = 0.868, I^2^ = 93%), and 3-year TBIL levels (*p* = 0.451, I^2^ = 82%).

**Figure 4:**
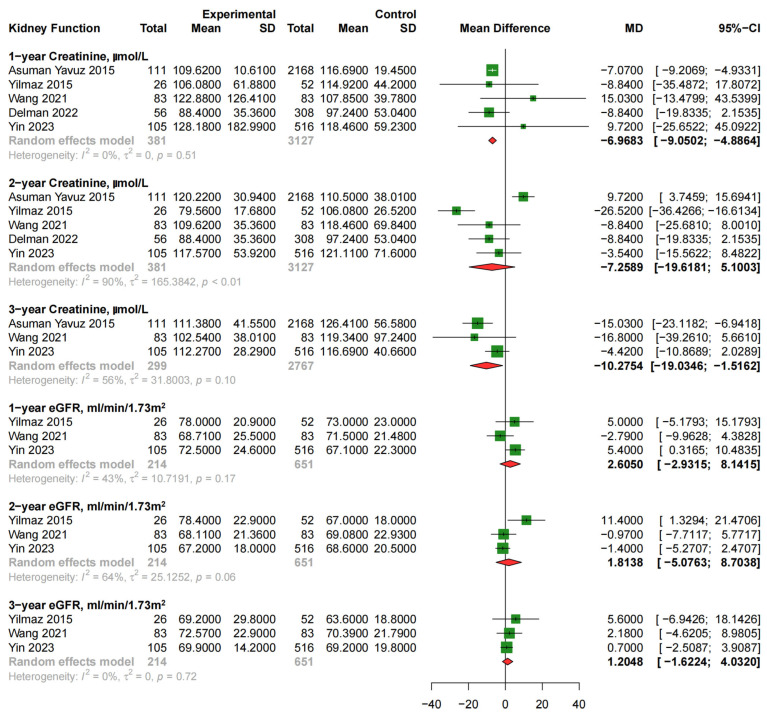
Comparison of kidney function of HBsAg− recipients between utilizing HBsAg+ kidneys and utilizing HBsAg− kidneys [12,15,16,37,38]. eGFR: estimated glomerular filtration rate.

Regarding kidney function, utilizing HBsAg+ kidneys had lower 1-year (*p* < 0.001, I^2^ = 0%) and 3-year serum creatinine (*p* = 0.021, I^2^ = 56%) but similar 2-year creatinine levels (*p* = 0.250, I^2^ = 90%) (Figure 4). eGFR levels were similar between groups at 1 year (*p* = 0.356, I^2^ = 43%), 2 year (*p* = 0.606, I^2^ = 64%), and 3 year (*p* = 0.404, I^2^ = 0%).

### 3.8. HBV-Related Complications

**Figure 5:**
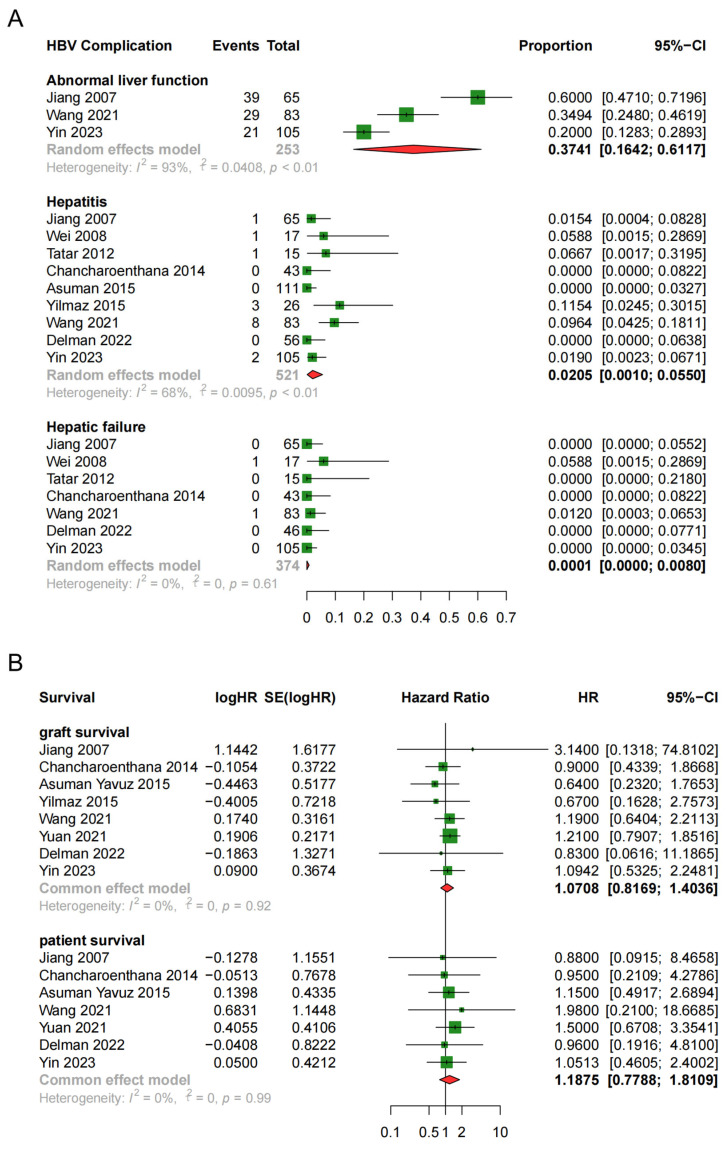
(**A**) Incidence of HBV complications: abnormal liver function; hepatitis; hepatic failure [12,15,16,31,32,34,36,37,38]. (**B**) graft and patient survival of recipients receiving HBsAg+ kidneys compared with those receiving HBsAg− kidneys [12,13,15,16,32,36,37,38].

The incidence of abnormal liver function was evaluated in three studies (89/253 [35.18%]) with high heterogeneity (I^2^ = 97%) [14,32,38] (Figure 5A). The incidence of hepatitis was assessed in nine studies (16/521 [3.07%]) with high heterogeneity (I^2^ = 68%) [12,14,15,16,31,32,34,36,38]. Additionally, seven studies evaluated the incidence of hepatic failure (2/237 [0.84%]) with no heterogeneity (I^2^ = 0%) [12,14,31,32,34,36,38].

### 3.9. Graft Survival

After receiving HBsAg+ kidneys, the 1-, 3-, 5-, and 10-year graft survival rates were 96.6%, 93.8%, 85.2%, and 82.7% (Table 3). After receiving HBsAg− kidneys, the 1-, 3-, 5-, and 10-year graft survival rates were 96.5%, 92.1%, 88.3%, and 85.6%, respectively. Further analyses showed that recipients receiving HBsAg+ kidneys and those receiving HBsAg− kidneys had similar graft survival rates (HR: 1.07, 95%CI: 0.82–1.40, *p* = 0.620) without heterogeneity (I^2^ = 0%) (Figure 5B). A contour-enhanced funnel plot is shown in Figure S6, and no publication bias was revealed in the Egger test (*p* = 0.456).

### 3.10. Patient Survival

After receiving HBsAg+ kidneys, the pooled 1-, 3-, 5-, and 10-year patient survival rates were 97.8%, 97.1%, 93.0% and 91.4%, respectively (Table 3). After receiving HBsAg− kidneys, the pooled 1, 3-, 5-, and 10-year patient survival rates were 98.3%, 97.1%, 95.6%, and 93.9%, respectively. Further comparison showed that HBsAg− recipients accepting HBsAg+ kidneys had a similar patient survival (HR:1.19, 95%CI: 0.78–1.81, *p* = 0.425) to those receiving HBsAg− kidneys without heterogeneity (I^2^ = 0%) (Figure 5B). A contour-enhanced funnel plot is shown in Figure S6, and no publication bias was revealed in the Egger test (*p* = 0.713).

## 4. Discussion

To the best of our knowledge, this is the first meta-analysis to summarize the risk of donor-derived HBV infection. Although our results showed that utilizing HBsAg+ kidneys had comparable liver function, kidney graft function, and survival rates, we report the potential risk of donor-derived HBV infection, hepatitis, and hepatic failure. Further, we revealed that HBV DNA+ donors, HBsAb− recipients, and HBcAb− recipients were potential risk factors of HBV transmission. These findings may help evaluate the utilization of HBsAg+ kidneys, facilitate a more informed consent process, and provide guidance for establishing the optimal antiviral prophylaxes against HBV infection.

Overall, the incidence of donor-derived HBV infection was low, which can be attributed to several factors. First, the concentration of HBV in the kidney is significantly lower than in the liver [39,40]. After kidney procurement, ex vivo perfusion would further eliminate HBV virions from the fluids [41]. Second, after transplantation, HBsAb can effectively bind to HBsAg, preventing the virus from attaching to and entering hepatocytes, thus blocking the establishment of productive infection [42]. Third, the administration of HBIG and/or posttransplant antiviral prophylaxes further contributes to reducing the risk of transmission; however, the type, timing, and duration of these prophylactic strategies varied substantially across studies, which may have influenced the observed outcomes.

As suggested, HBV immunity, acquired through natural exposure or vaccination against HBV, was necessary for HBsAg− recipients receiving HBsAg+ kidney transplantation [5,6]. In our current meta-analysis, we report that HBV infection risk was hierarchically corrected with HBsAb levels, with HBsAb < 10 IU/L (HBsAb−) having higher risk and HBsAb > 100 IU/L having lower risk. During the informed consent process, the transplant team can provide more proactive advice for HBsAb+ recipients, emphasizing the low risk of transmission and excellent survival outcomes. Also, accepting HBsAg+ kidneys would significantly reduce the timing on the waitlist. Delman et al. reported that recipients receiving HBV NAT+ organs experienced significantly less time on the waitlist than recipients of HBV NAT− organs (283.4 days vs. 424.2 days, *p* < 0.01) [12].

Notably, approximately 30–70% of patients undergoing dialysis had negative HBsAb due to impaired immune responses [43,44]. Despite efforts to enhance HBV vaccination response rate, including increasing the vaccine doses or adding adjuvants, a substantial number of patients continued to remain HBsAb− [45]. In such cases, it becomes essential for transplant teams to engage in comprehensive discussions with patients about the potential risks and benefits of receiving HBsAg+ kidneys. In certain urgent situations (e.g., severe heart failure), where the benefits of timely transplantation outweigh the risk of acquiring HBV infection and when an HBsAg+ donor is the only available option, receiving an HBsAg+ kidney transplantation may be considered. For these non-immune recipients, passive immunity by receiving HBIG and posttransplant antiviral prophylaxes would play a crucial role in reducing the HBV infection risk.

Previous studies required that the HBsAg+ donor have undetectable HBV DNA [16,35]. When HBV DNA coexists with an HBsAg+ person, the infectivity of the virus increases. National population-based epidemiological studies have reported that the prevalence of positive HBV DNA can be as high as 50.0% in HBsAg carriers, particularly in endemic areas [46]. Declining these donors would significantly decrease the donor pool. In our current meta-analysis, we observed a higher risk of donor-derived HBV transmission utilizing HBV DNA+ kidneys. These findings indicated that reducing HBV DNA levels before kidney donation could potentially mitigate the risk of HBV transmission. For living donors, antiviral treatment prior to donation may be considered as the optimal choice to achieve undetectable HBV DNA levels [38]. For kidneys procured from deceased donors, it is hypothesized that ensuring sufficient ex vivo perfusion during kidney procurement might help physically reduce intra-graft HBV DNA levels by flushing out residual viral particles [41]. Further studies are needed to explore the duration of antiviral treatment in living donors and the perfusion volume and time for kidneys from deceased donors.

In our study, although the incidence of hepatitis was low, we observed a relatively high incidence of abnormal liver function. In kidney transplant recipients, abnormal liver function can be attributed to the use of immunosuppressive drugs, the use of antibacterial drugs, and infectious diseases. Hepatotoxicity attacks have been reported in 10–30% of kidney recipients. Our results reported a higher incidence of abnormal liver function, which may be associated with HBV exposure in recipients receiving HBsAg+ kidneys. We did report that utilizing HBsAg+ had higher 1-year ALT levels. The most severe complication of HBV infection is hepatic failure, which has been reported at 0.1–0.5% among all patients with acute HBV infection [47]. In our current results of 21 infected patients, 2 experienced hepatic failure, which is numerically higher.

Notably, we observed higher incidence of HBcAb, which represents resolved HBV infection after HBV exposure. While HBsAg is the marker of chronic infection and indicates the greatest risk for liver complications, recipients who are HBsAg− but HBcAb+ have intrahepatic virus present and are at risk for HBV reactivation, especially under immunosuppressive conditions [48]. A recent meta-analysis reported that the overall HBV reactivation incidence was 2.5% in kidney transplant recipients who were HBcAb-positive [22]. This risk was found to be higher in recipients who received lymphocyte-depleting agents and those with negative HBsAb. In the context of HBsAg+ kidney transplantation, transmission of HBV can be documented by conversion to HBsAg or HBcAb positivity. However, it should be emphasized that HBcAb conversion alone, in the absence of HBsAg or HBV DNA, represents controlled or resolved exposure rather than active productive infection requiring antiviral therapy. The distinction is critical for clinical management and outcome reporting. Considering relatively low incidence of HBV infection, HBcAb conversion rate may also be used as a surrogate endpoint to evaluate HBV transmission risk and explore potential risk factors in future prospective studies.

According to the American Society of Transplantation Consensus Guidelines for recipient management for solid organ transplantation from HBV+ donors [19], standardized antiviral prophylaxis protocols have not been established. Based on our results, donor and recipient HBV serology was associated with the risk of HBV infection. Hence, we established a stratified prophylaxis regimen, including donor antiviral treatment, recipient HBV vaccination, and posttransplant antiviral prophylaxis. If the recipients were HBsAb-, they would receive a single-dose of 2000 IU HBIG and Entecavir for 3 months. If the recipient had HBsAb level at 10–100 IU/L, they would receive a single-dose of 2000 IU HBIG and Entecavir for 1 month. If the recipient had HBsAb above 100 IU/L and the donor had negative HBV DNA, the recipient would not receive any antiviral prophylaxis. A multicenter prospective study has been launched to evaluate the safety and efficacy of this stratified prophylactic regimen (NCT04562051) [49].

The observed heterogeneity carries important clinical implications that warrant explicit discussion. First, the wide variation in HBV transmission rates (0–30%) indicates that the pooled estimate cannot be directly extrapolated to all transplant centers. Clinicians must interpret this summary value in the context of their local antiviral prophylaxis protocols (e.g., Entecavir/Tenofovir vs. Lamivudine), donor HBV-DNA status, and recipient serological background (e.g., HBcAb positivity). Our subgroup analyses suggest that using high-barrier antiviral drugs significantly reduces transmission risk, which should inform clinical decision-making. Second, heterogeneity in liver dysfunction rates likely reflects inconsistent diagnostic criteria (e.g., ALT upper limit ranging from 40 to 60 U/L) and competing etiologies (e.g., drug-induced liver injury and CMV infection). Future studies should adopt standardized definitions to improve comparability. Third, the persistence of heterogeneity after adjusting for available covariates via meta-regression suggests that unmeasured factors (e.g., adherence to antiviral therapy, donor age, and cold ischemia time) contribute to outcome variability. Therefore, the pooled effect sizes presented in this meta-analysis should be regarded as historical benchmarks reflecting a range of clinical practices, rather than precise risk estimates for a given patient. Individualized risk assessment remains essential.

Several limitations should be emphasized in the present meta-analysis. First, most of the included studies were retrospectively designed. A retrospective design may have underestimated the incidence of HBV infection due to the lack of routine HBV monitoring. Second, laboratory tests for monitoring HBV serology have changed and had higher sensitivity and specificity over time. Hence, the incidence of HBV infection may have been underestimated in the early published studies. Third, several early published studies were designed as single-arm studies without a control group. For these studies, the estimated OR/HR using HBV infection as the endpoint may be not accurate enough. Additionally, the potential misclassification between de novo donor-derived infection and reactivation in HBcAb-positive recipients could not be fully addressed due to limited individual patient data. Future studies should adopt standardized criteria to distinguish isolated HBcAb conversion from active infection and to differentiate reactivation from new infection in HBcAb-positive recipients. Fourth, follow-up duration was not reported in several studies and also varied. A short follow-up time may not be sufficient to demonstrate the effectiveness of using HBsAg + kidneys, while a long follow-up time may underestimate the incidence of HBV in the absence of continuous monitoring. Fifth, the NOS score varied. Including low-quality studies may introduce bias and reduce the reliability of the current findings. Last, high heterogeneity has been observed. This resulted from the differences in patient baseline characteristics based on meta regression analysis, and therefore, subgroup analyses have been conducted. Considering these limitation, prospective studies with larger sample sizes and standardized monitoring protocols are needed to provide more robust evidence. Finally, the time interval between the systematic search (August 2023) and the final analysis (early 2025) may introduce a risk of missing very recent evidence. However, as detailed in the Methods section, a targeted re-screen before submission did not identify new studies capable of affecting our main conclusions. Nonetheless, readers are advised to interpret the results with consideration of this temporal limitation.

## 5. Conclusions

Although the current study reveals encouraging but preliminary outcomes after kidney transplantation from HBsAg+ donors to HBsAg− recipient, the risk of donor-derived HBV infection and HBV complications should be evaluated, warranting cautious clinical application. Our findings might have several implications for clinical practice. Physicians and patients can better understand the potential risks and benefits of accepting HBsAg+ kidneys, improving the informed consent process. The current findings also supported utilizing HBsAg+ kidneys, which might help expand the donor pool. Additionally, these results might provide valuable evidence for establishing standardized antiviral prophylactic protocols, which would further enhance the safety of using HBsAg+ kidneys.

## Figures and Tables

**Table 1:** Baseline characteristics of 16 included studies.

Author Year	Country	Study Design	Sample Size	Control Group	Recipients Age	Male, %	Follow-Up	HBsAb Status	HBcAb Status	No. of HBV Infection	Timing of HBV Infection	Antiviral Prophylaxis	Outcome
Bedrossian 1993 [28]	France	Retrospective	8	No	NA	NA	12–24 months	unreported	unreported	0	/	HBIG	incidence of HBsAg
Turc Baron 1997 [29]	France	Retrospective	7	No	52.6	4/7	unreported	7 > 100 IU/L	unreported	0	/	HBIG	incidence of HBsAg
Berber 2005 [30]	Turkey	Retrospective	7	No	unreported	unreported	mean: 42.6	7 HBsAb+	unreported	0	/	Lamivudine	incidence of HBsAg
Wei 2008 [31]	China	Retrospective	17	No	34	9/21	mean: 43.4 months	unreported	unreported	2	8 months, unknown	None	incidence of HBsAg, 5-year patient and graft survival
Jiang 2007 [32]	China	Prospective	65	Yes	40.0	45/65	mean: 38.7 months	65 HBsAb+	33 HBcAb+	1	3 months	HBIG, Lamivudine	incidence of HBsAg, 1-year/3-year/5-year graft and patient survival, acute rejection
Sumethkul 2009 [33]	Thailand	Retrospective	14	No	43.3	unreported	mean: 137 months	14 HBsAb+	unreported	0	/	None	incidence of HBsAg, 1-year/5-year/10-year graft and patient survival
Tatar 2012 [34]	Turkey	Retrospective	15	No	43.3	unreported	mean: 40 months	15 HBsAb+	unreported	0	/	HBIG, Lamivudine	incidence of HBsAg
Tuncer 2012 [35]	Turkey	Retrospective	35	No	42.2	20/35	Unreported	35 HBsAb+	unreported	0	/	None	incidence of HBsAg, graft loss, patient death, 1-year/2-year creatinine/eGFR/AST/ALT
Chancharoenthana 2014 [36]	Thailand	Retrospective	43	Yes	50.7	23/43	median: 58.2 months	43 HBsAb > 100 IU/L	13/43 HBcAb+	0	/	HBIG, Lamivudine, None	incidence of HBsAg, 1-year/3-year/5-year/10-year graft and patient survival
Asuman Yavuz 2015 [16]	Turkey	Retrospective	111	Yes	43.1	75/111	unreported	111 HBsAb+	unreported	0	/	None	incidence of HBsAg, 1-year/2-year/3-year creatinine/AST/ALT, graft loss, patient death, acute rejection
Yilmaz 2015 [15]	Turkey	Retrospective	26	Yes	37	61.5%	Unreported	unreported	unreported	3	Unreported	Lamivudine	incidence of HBsAg, 1-year/3-year/5-year graft and patient survival, rejection, DGF, CMV infection
Wang 2021 [14] *	China	Retrospective	83	Yes	50	47%	median: 38.3 months	63 HBsAb+	58 HBcAb+	2	6 months, 12 months	HBIG, Lamivudine, Entecavir	incidence of HBsAg, 1-year, 2-year, 5-year graft and patient survival, graft loss, patient death, rejection
Yuan 2021 [13]	USA	Retrospective	144	Yes	52.3	62.5%	Unreported	unreported	unreported	/	/	Unreported	1-year, 3-year, 5-year, 10-year graft and patient survival
Delman 2022 [12]	USA	Prospective	56	Yes	57.1	69.6%	median: 183 days	unreported	unreported	9	Median: 185 days	Entecavir	incidence of HBsAg+, 1-year, 3-year, 5-year graft and patient survival
Wang 2022 [37] *	China	Retrospective	83	Yes	50	47%	median: 38.3 months	63 HBsAb+	58 HBcAb+	2	6 months, 12 months	HBIG, Lamivudine, Entecavir	incidence of HBsAg, 1-year/2-year/3-year creatinine/AST/SLT
Yin 2023 [38]	China	Retrospective	105	Yes	35.6	81.0%	mean: 23.0 months	80 HBsAb+	62 HBcAb+	4	1 month, 2 month, 8 month, 17 month	HBIG, Lamivudine, Entecavir, Tenofovir	incidence of HBsAg, graft loss, patient death, 1-year/2-year creatinine/eGFR/AST/ALT

HBsAb: antibody against hepatitis B surface antigen; HBcAb: antibody against hepatitis B core antigen; HBIG: hepatitis B immunoglobulin; HBsAg: hepatitis B surface antigen; ALT: alanine aminotransferase; AST: aspartate aminotransferase; TBIL: total bilirubin; eGFR: estimated glomerular filtration rate. *: The same population was used in the two studies.

**Table 2:** Meta regression of the incidence of HBV infection.

Characteristics	OR (95%CI)	*p* Value
Age	1.001 (0.989–1.012)	0.904
Sex, Male vs. Female	1.034 (0.463–2.311)	0.935
Publication Year	1.004 (0.994–1.013)	0.477
Region, Asian vs. non-Asian	1.195 (1.016–1.406)	0.032
Study design, retrospective vs. prospective	0.882 (0.745–1.045)	0.147
Sample size	0.999 (0.997–1.001)	0.432
Donor HBV DNA status, − vs. +	0.865 (0.766–0.976)	0.019
Donor type, living vs. deceased	0.922 (0.789–1.077)	0.306
Recipient HBsAb status, + vs. −	0.510 (0.374–0.695)	<0.001
Recipient HBcAb status, − vs. +	0.750 (0.484–1.163)	0.199
Antiviral prophylaxis, Yes vs. No	1.018 (0.876–1.184)	0.814
Follow-up duration	0.998 (0.996–1.000)	0.078

HBsAb: antibody against hepatitis B surface antigen; HBcAb: antibody against hepatitis B core antigen; HBV: hepatitis B virus.

**Table 3:** Graft survival and patient survival after kidney transplantation from HBsAg+ or HBsAg− donors into HBsAg− recipients.

Survival (% An 95%CI)	HBsAg+ Donor			HBsAg− Donor		
	No. Studies	Sample Size	Survival (95%CI)	No. Studies	Sample Size	Survival (95%CI)
1-year graft survival	7	431	96.6% (94.2–98.5%)	7	79,539	96.5% (94.0–98.4%)
3-year graft survival	5	361	93.8% (87.9–98.0%)	5	79,204	92.1% (86.2–96.5%)
5-year graft survival	6	378	85.2% (73.2–94.2%)	5	79,204	88.3% (80.8–94.2%)
10-year graft survival	2	69	82.7% (72.6–91.0%)	2	138	85.6% (79.1–91.1%)
1-year patient survival	7	431	97.8% (95.8–99.3%)	7	79,539	98.3% (96.7–99.4%)
3-year patient survival	5	361	97.1% (92.3–99.8%)	5	79,204	97.1% (93.1–99.5%)
5-year patient survival	6	378	93.0% (86.5–97.7%)	5	79,204	95.6% (89.2–99.3%)
10-year patient survival	2	69	91.4% (83.2–97.3%)	2	138	93.9% (64.9–100%)

HBsAg: hepatitis B surface antigen.

## Data Availability

After publication, the data and R code will be made available as Supplementary Materials.

## Supplementary Materials

The following supporting information can be downloaded at https://www.mdpi.com/article/10.3390/jcm15156048/s1, Table S1: Search strategy. Table S2: The literature with reasons for exclusion after screening the full-text. Table S3: Quality assessment of included 16 studies. Figure S1: Sensitivity analysis by sequentially removing individual study to evaluate the robustness of the pooled estimates. Figure S2: Contour-enhanced funnel plot of incidence of HBV infection. Figure S3: Subgroup analyses of pooled incidence of HBV infection after kidney transplantation using HBsAg+ donors. Figure S4: The timing and antiviral prophylaxes of recipients infected with HBV after transplantation utilizing HBsAg+ kidneys. Among 21 infected recipients, the timing of HBV infection was not reported in 3 recipients, and therefore, 18 recipients were included for the analysis. X axis: the duration of antiviral prophylaxes after transplantation; Y axis: the timing of HBV infection. Figure S5: Pooled incidence of HBcAb and HBsAb after kidney transplantation using HBsAg+ donors. Figure S6: Contour-enhanced funnel plot. A: Graft survival; B: patient survival. File S1. PRISMA 2020 Checklist.

## Abbreviations

HBV: hepatitis B virus; HBsAg: hepatitis B surface antigen; KDIGO: Kidney Disease: Improving Global Outcomes; KT: kidney transplantation; CI: confidence interval; OR: odds ratio; HR: hazards ratio; ALT: alanine aminotransferase; AST: aspartate aminotransferase; TBIL: total bilirubin; eGFR: estimated glomerular filtration rate.
