# Supplementary material for: Long-Term Clinical Outcomes of Kidney Transplantation from Hepatitis B Surface Antigen (HBsAg)-Positive Donors to HBsAg-Negative Recipients: A Systematic Review and Meta-Analysis of Real-World Studies
Source: J Clin Med. 2026 Aug 4;15(15):6048. doi: 10.3390/jcm15156048 (PMC13467147; doi:10.3390/jcm15156048)
Supplement: Supplementary file 1 [file jcm-15-06048-s001.zip › Figure S1-6.pdf]

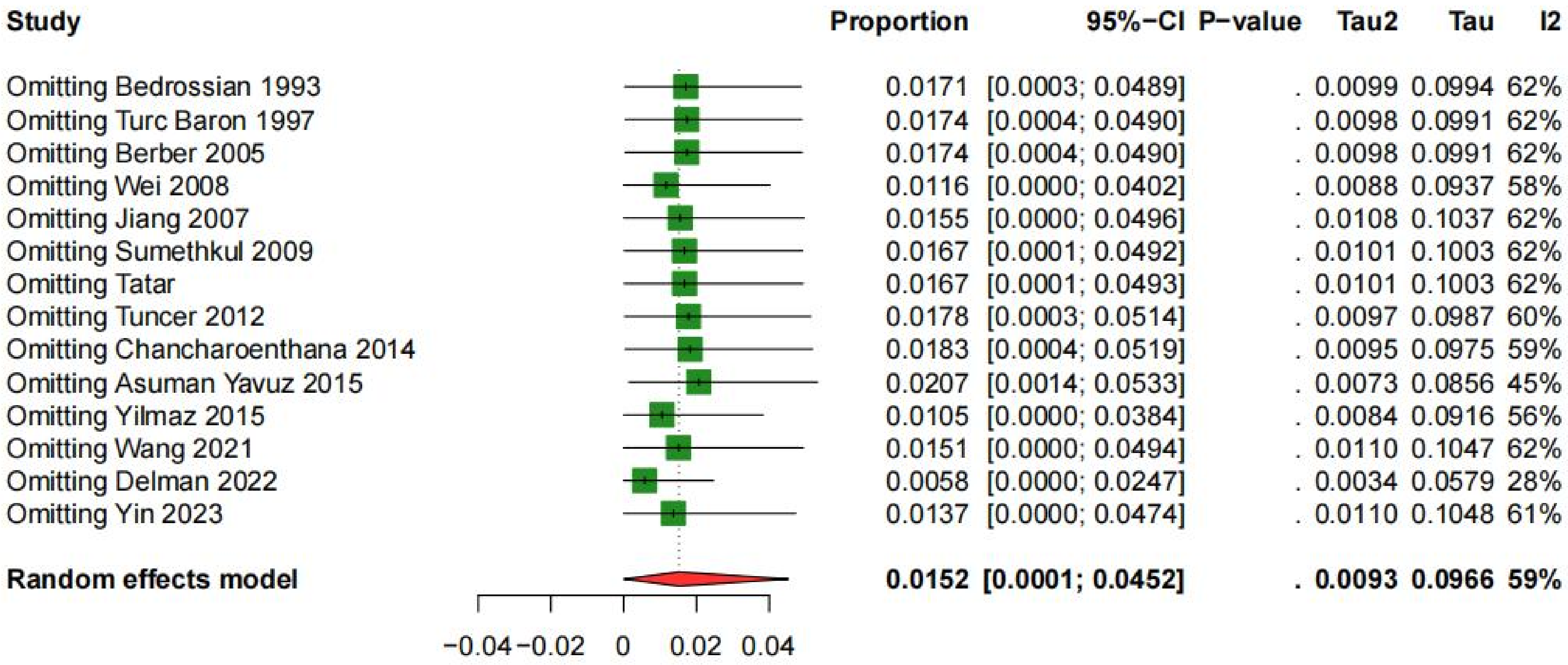

Figure S1: Sensitivity analysis by sequentially removing individual study to evaluate the robustness of the pooled estimates.

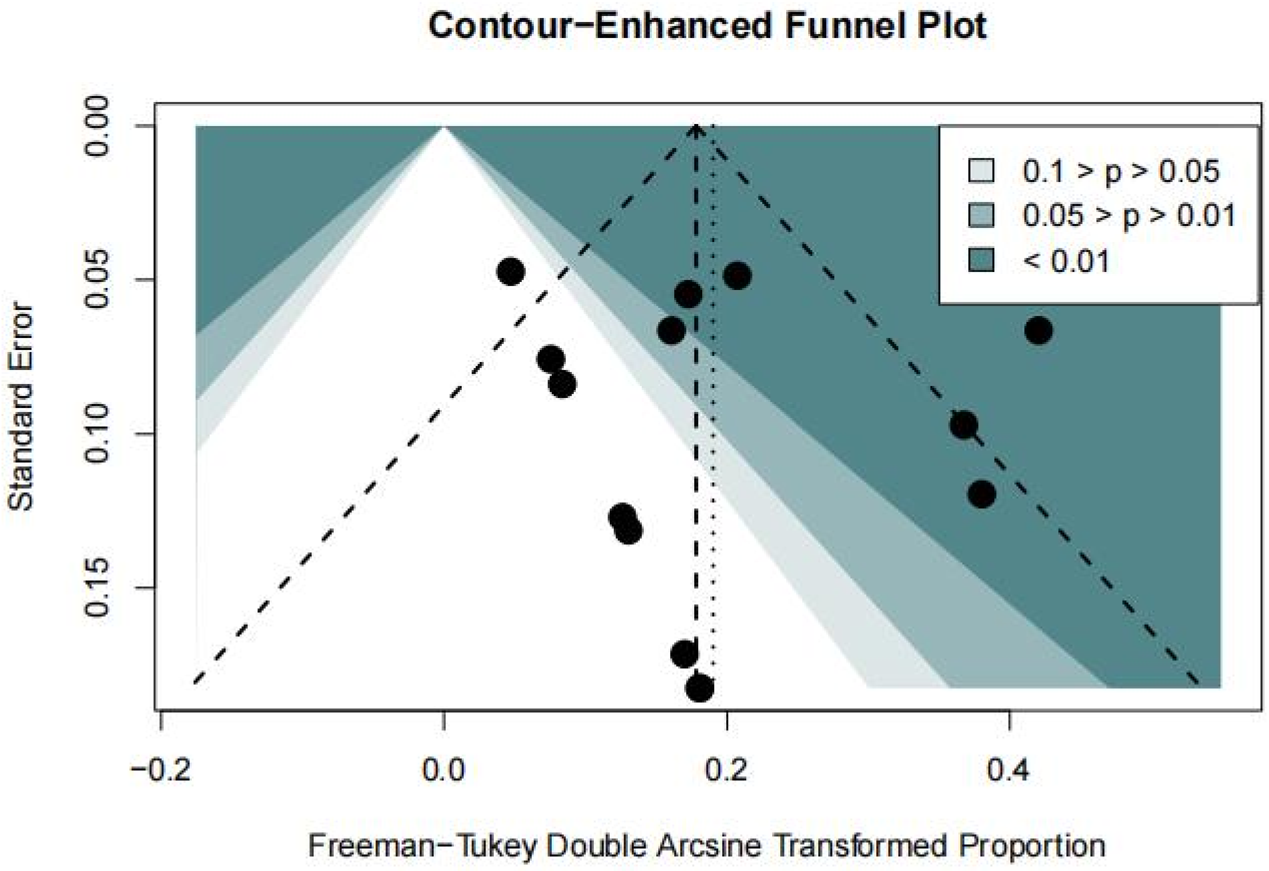

Figure S2: Contour-Enhanced Funnel Plot of incidence of HBV infection.

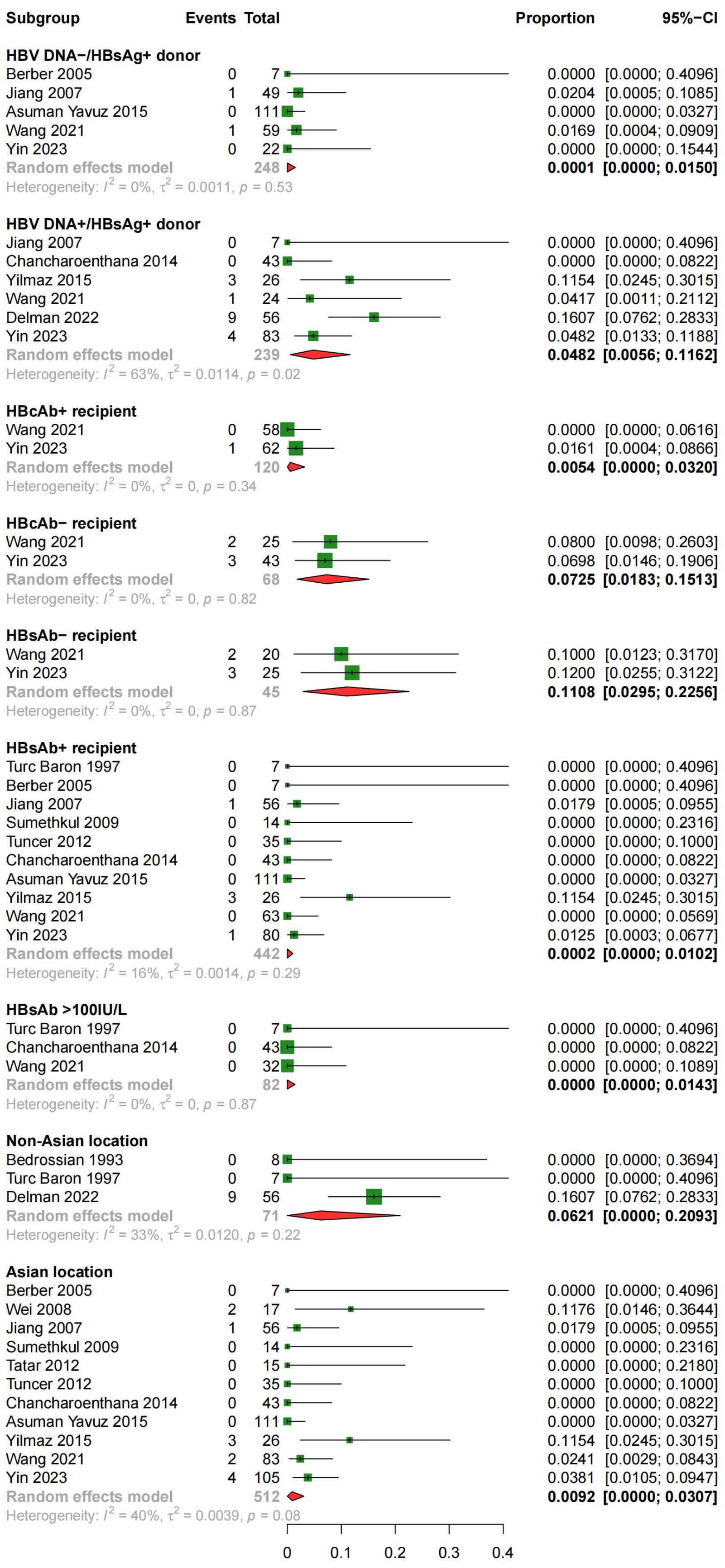

Figure S3: Subgroup analyses of pooled incidence of HBV infection after kidney transplantation using HBsAg+ donors.

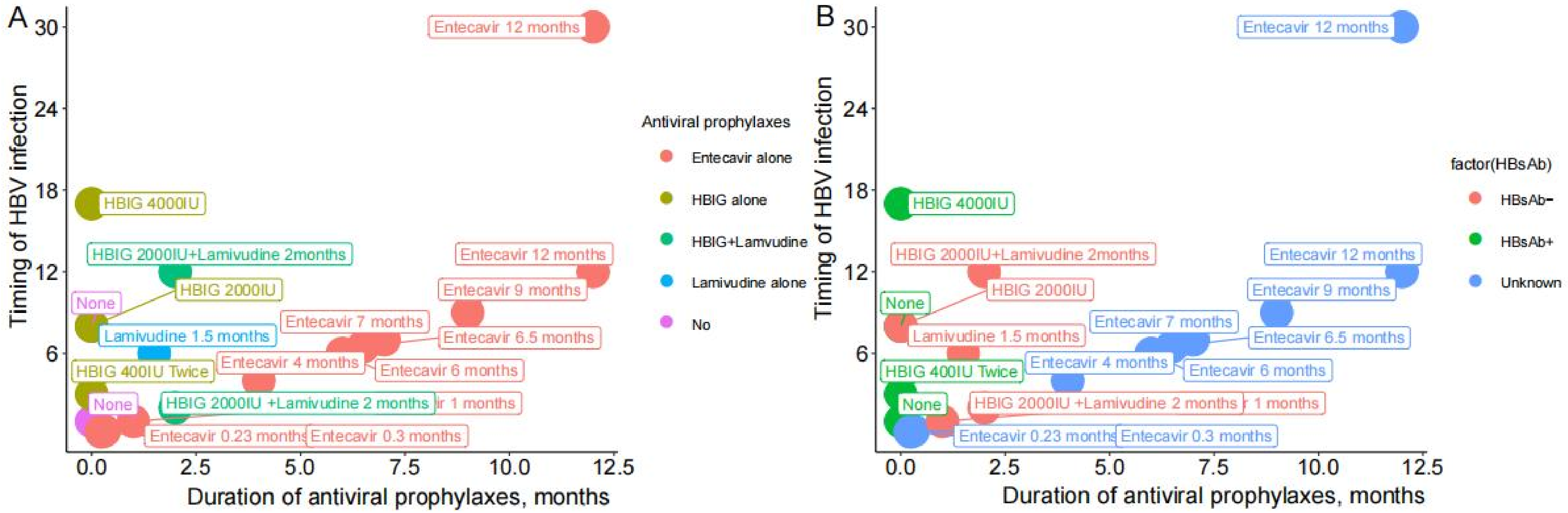

Figure S4: The timing and antiviral prophylaxes of recipients infected with HBV after transplantation utilizing HBsAg+ kidneys. Among 21 infected recipients, the timing of HBV infection was not reported in 3 recipients, and therefore 18 recipients were included for the analysis. X axis: the duration of antiviral prophylaxes after transplantation; Y axis: the timing of HBV infection.

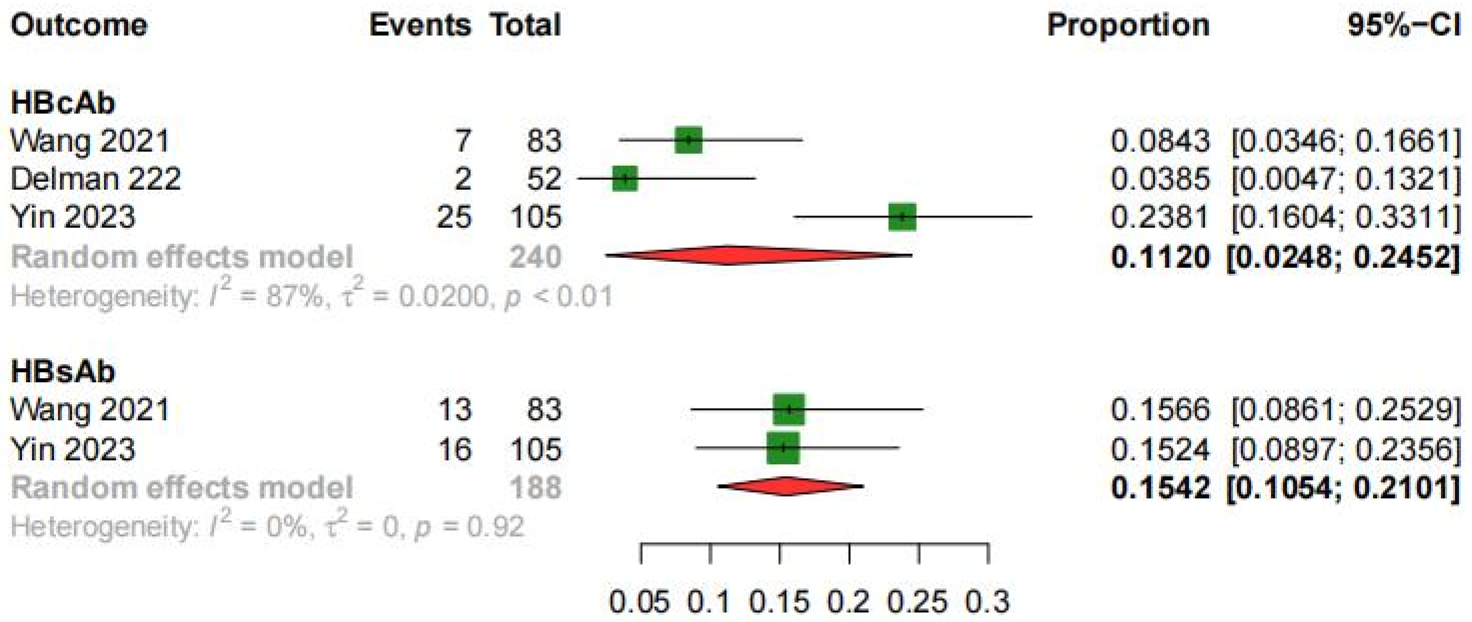

Figure S5: Pooled incidence of HBcAb and HBsAb after kidney transplantation using HBsAg+ donor

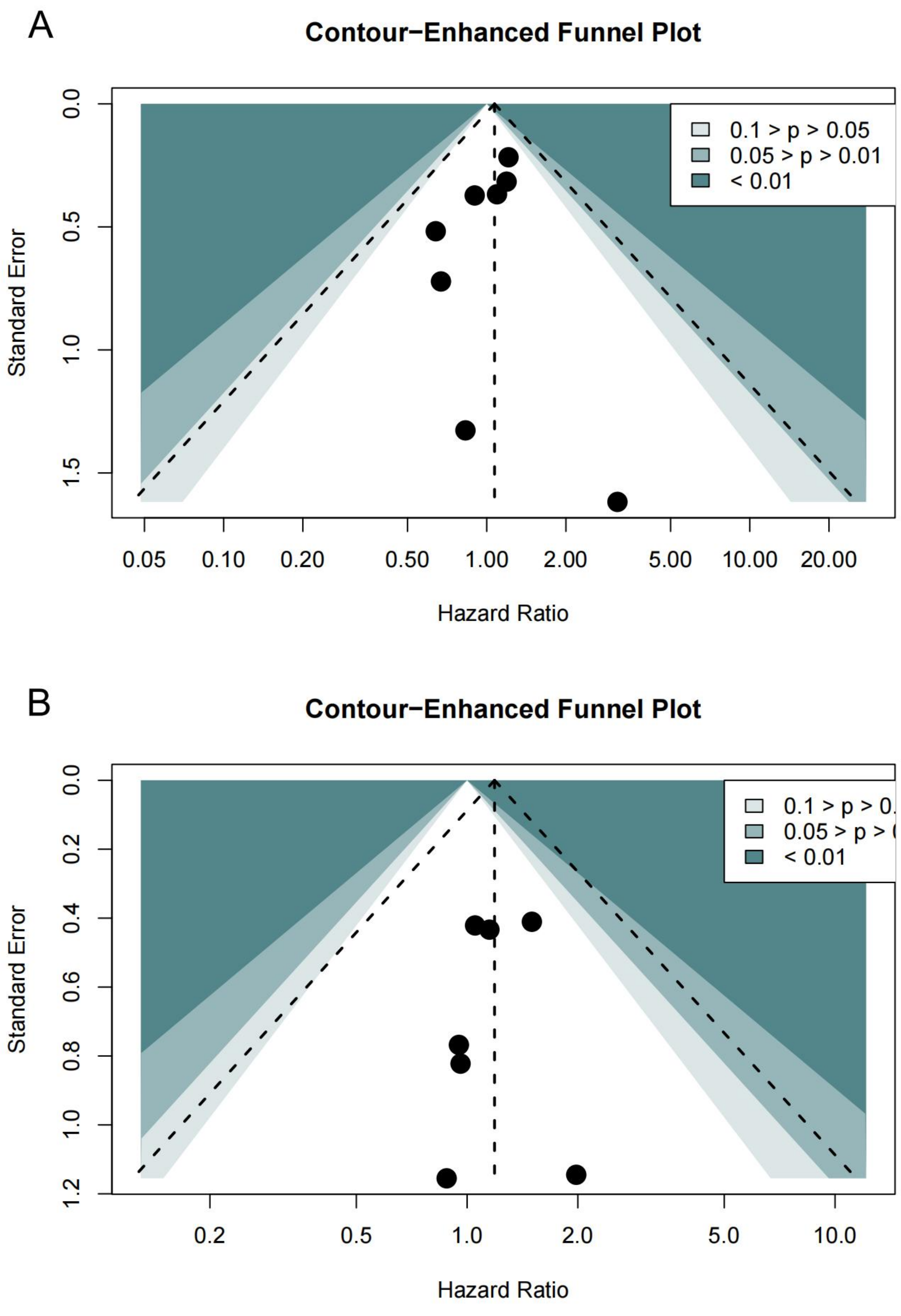

Figure S6: Contour-Enhanced Funnel Plot. A: graft survival; B: patient survival
