# Supplementary material for: Long-Term Clinical Outcomes of Kidney Transplantation from Hepatitis B Surface Antigen (HBsAg)-Positive Donors to HBsAg-Negative Recipients: A Systematic Review and Meta-Analysis of Real-World Studies
Source: J Clin Med. 2026 Aug 4;15(15):6048. doi: 10.3390/jcm15156048 (PMC13467147; doi:10.3390/jcm15156048)
Supplement: Supplementary file 1 [file jcm-15-06048-s001.zip › Table S1-3.pdf]

Table S1: Search Strategy

| Embase | | Pubmed | |
|---|---|---|---|
| 1 | exp hepatitis B virus/or exp hepatitis B/ | 1 | "Kidney Transplantation"[Title/Abstract] OR "renal transplantation"[Title/Abstract] OR "kidney graft"[Title/Abstract] OR "kidney donor"[Title/Abstract] OR "kidney transplant"[Title/Abstract] OR "renal transplant"[Title/Abstract] OR "Kidney Transplantation"[MeSH Terms] OR "Solid Organ Transplantation"[Title/Abstract] |
| 2 | exp hepatitis B surface antigen/ | 2 | HBV[Title/Abstract] OR HBsAg[Title/Abstract] OR HBsAb[Title/Abstract] OR HBcAb[Title/Abstract] OR anti-HBs[Title/Abstract] OR anti-HBc[Title/Abstract] OR HBeAg[Title/Abstract] OR "Hepatitis B"[MeSH Terms] OR "hepatitis b, chronic"[MeSH Terms] OR "Hepatitis B Surface Antigens"[MeSH Terms] OR "Hepatitis B virus"[MeSH Terms] OR "Hepatitis B e Antigens"[MeSH Terms] OR "Hepatitis B Core Antigens"[MeSH Terms] OR "Hepatitis B Antibodies"[MeSH Terms] |
| 3 | exp hepatitis B surface antibody/ | 3 | 1 AND 2 |
| 4 | exp hepatitis B core antibody/ | | |
| 5 | exp "hepatitis B(e) antigen"/ | | |
| 6 | exp "hepatitis B(e) antibody"/ | | |
| 7 | exp kidney transplantation/ | | |
| 8 | exp kidney graft/ | | |
| 9 | 1 or 2 or 3 or 4 or 5 or 6 | | |
| 10 | 7 or 8 | | |
| 11 | 9 and 10 | | |

Table S2: The literature with reasons for exclusion after screening the full-text.

| Publications | Reasons |
|---|---|
| Lai M.K., Huang C.C.,Chu S.H., et al. The outcome of kidney transplantation from HBsAg-positive donors. Asian Journal of Surgery 1994;4:340-343 | HBsAg+ donors to HBsAg+ recipients |
| Santos AF, Busetto F, Franco R, et al. Renal transplantation using hepatitis B or C serology-positive donors. Transplant Proc 1997;29:3320. | HBsAg+ donors to HBsAg+ recipients |
| Kim JA, Huh W, Lee KW, et al. Cadaveric renal transplantation in hepatitis B antigen-positive recipients using hepatitis B antigen-positive donor organs with lamivudine treatment. Transplant Proc 2004;36:1434-7. | HBsAg+ donors to HBsAg+ recipients |
| Veroux M, Puliatti C, Macarone M, et al. Kidney transplantation from hepatitis B surface antigen-positive donors into hepatitis B surface antigen-positive recipients: preliminary findings. Transplant Proc 2005;37:2467-8. | HBsAg+ donors to HBsAg+ recipients |
| Russo FP, Viganò M, Stock P, et al. HBV-positive and HIV-positive organs in transplantation: A clinical guide for the hepatologist. J Hepatol 2022;77:503-515. | Reviews |
| Pilmore HL, Gane EJ. Hepatitis B-positive donors in renal transplantation: increasing the deceased donor pool. Transplantation 2012;94:205-10. | Reviews |
| Khemichian S, Kahn J, Terrault NA. Use of Hepatitis B Virus-Positive Organs in Organ Transplantation. Clin Liver Dis. 2021;25:841-857. | Reviews |

| | |
|---|---|
| Chan MK, Chang WK. Renal transplantation from HBsAg positive donors to HBsAg negative recipients. BMJ 1988; 297: 522–523 | Sample size <5 |
| al-Khader AA, Dhar JM, al-Sulaiman M, et al. Renal transplantation from HBsAg positive donors to HBsAg negative recipients. BMJ 1988; 297: 854. | Sample size <5 |
| Chan PC, Lok AS, Cheng IK,et al. The impact of donor and recipient hepatitis B surface antigen status on liver disease and survival in renal transplant recipients. Transplantation 1992; 53: 128–131. | Sample size <5 |
| Okamoto M, Yoshimura N, Nakai I, et al. Kidney transplantation from a hepatitis B surface antigen-positive donor to a HBSAG-negative recipient. Transplant Proc 1999;31:2869. | Sample size <5 |
| Mohrbach J, JanssenMW,Heine GH, et al. Successful outcome of kidney transplantation from a HBV-DNA positive donor into recipients with cleared HBV-infection using a pre-emptive therapy approach. J Clin Virol 2010; 49: 53–57. | Sample size <5 |
| Singh G, Hsia-Lin A, Skiest D, et al. Successful kidney transplantation from a hepatitis B surface antigen-positive donor to an antigen-negative recipient using a novel vaccination regimen. Am J Kidney Dis 2013; 61: 608–611. | Sample size <5 |

Table S3: Quality assessment of included 16 studies.

| Author Year | Total NOS score | Selection (4 score) | | | | Comparability (2 score) | Outcome (3 score) | | |
|---|---|---|---|---|---|---|---|---|---|
| | | Representativeness of the exposed cohort (1 score) | Selection of the non-exposed cohort (1 score) | Ascertainment of exposure (1 score) | Outcomes were not present at study initiation (1 score) | Comparability of cohorts on the basis of the design or analysis | Assessment of outcome of follow-up (1 score) | Was follow-up long enough for outcome to occur **(**1 score**)** | Adequacy (1 score) |
| Bedrossian 1993 | 5 | 1 | 0 | 1 | 1 | 0 | 1 | 1 | 0 |
| Turc Baron 1997 | 5 | 1 | 0 | 1 | 1 | 0 | 1 | 1 | 0 |
| Berber 2005 | 5 | 1 | 0 | 1 | 1 | 0 | 1 | 1 | 0 |
| Wei 2008 | 5 | 1 | 0 | 1 | 1 | 0 | 1 | 1 | 0 |
| Jiang 2007 | 9 | 1 | 1 | 1 | 1 | 2 | 1 | 1 | 1 |
| Sumethkul 2009 | 5 | 1 | 0 | 1 | 1 | 0 | 1 | 1 | 0 |
| Tatar 2012 | 6 | 1 | 0 | 1 | 1 | 1 | 1 | 1 | 0 |
| Tuncer 2012 | 5 | 1 | 0 | 1 | 1 | 0 | 1 | 1 | 0 |
| Chancharoenthana 2014 | 8 | 1 | 1 | 1 | 1 | 2 | 1 | 1 | 0 |
| Asuman Yavuz 2015 | 7 | 1 | 1 | 1 | 1 | 1 | 1 | 1 | 0 |
| Yilmaz 2015 | 7 | 1 | 1 | 1 | 1 | 1 | 1 | 1 | 0 |
| Wang 2021 | 8 | 1 | 1 | 1 | 1 | 1 | 1 | 1 | 1 |
| Yuan 2021 | 7 | 1 | 1 | 1 | 1 | 1 | 1 | 1 | 0 |
| Delman 2022 | 8 | 1 | 1 | 1 | 1 | 2 | 1 | 0 | 1 |
| Wang 2022 | 9 | 1 | 1 | 1 | 1 | 2 | 1 | 1 | 1 |
| Yin 2023 | 8 | 1 | 1 | 1 | 1 | 1 | 1 | 1 | 1 |
